# A parameter study of ultrasound assisted enzymatic esterification

**DOI:** 10.1038/s41598-022-05551-x

**Published:** 2022-01-26

**Authors:** Anamaria Vartolomei, Ioan Calinescu, Mircea Vinatoru, Adina Ionuta Gavrila

**Affiliations:** Department of Bioresources and Polymer Science, Faculty of Applied Chemistry and Material Science, University Politehnica of Bucharest, 1-7 Gh. Polizu Str., 011061 Bucharest, Romania

**Keywords:** Biocatalysis, Process chemistry

## Abstract

This work is focused on the study of the esterification parameters for the ultrasound assisted synthesis of isoamyl acetate catalyzed by lipase Lipozyme 435 in a continuous loop reactor. Investigating the influence of different parameters shows that a higher concentration of ester (462 mg/g mixture) can be obtained at a temperature of 50 °C, flow rate 0.16 mL/min. The best ultrasonication conditions are: sonication applied continuously for a short time (20 min), ultrasound power 32 mW and amplitude 20%. The enzyme can be successfully reused tree times without loss of enzyme activity. Reaction kinetics for isoamyl acetate ultrasound assisted production showed that satisfactory reaction concentration (close to the equilibrium concentrations) could be reached in short reaction times (2 h). Ultrasound assisted enzymatic esterification is consequently a cleaner and a faster process.

## Introduction

Biocatalysts, green, clean and efficient products, are increasingly used to replace chemical catalysts, especially due to their high specificity, regio- and stereo- selectivity in mild conditions, leading to a sustainable chemical process^1^. Enzymes, protein with catalytic activity, have been used in processes producing: biofuel^2^, food and beverage^3^, in research and biotechnologies^4,5^, in cleaning products^6^, for diagnostics^7^, animal feed^8^. Enzyme immobilization on a suitable support is a need to increase its reusability. In general, such a process (immobilization) reduces the efficiency of enzymes^9^ caused by the immobilization procedures and by mass-transfer limitations^10^. The uses of immobilized enzymes is still challenging due to their costs and storage problems^11^. However, due to the easiness of products recovery, purification and separation, immobilized enzymes are preferred over free enzymes^12^. For this reason, methods to maintain or activate the immobilized enzymes are of paramount importance.

There are several methods to overcome the current limitations related to immobilized enzymes, some of them being chemically binding^13^, the use of sub- and supercritical carbon dioxide for the enhancement of enzymatic activity and stability (case of α-amylase), multiple-site mutagenesis of lipase B *Candida Antarctica*^14,15^. Among these methods, ultrasound as a meaning to enhance the activity of enzymes is a very promising technique. Improving the activity of tyrosinase^16^, enzymatic hydrolysis of starch for better glucoamylase activity^17^, and dual frequency ultrasound enhancing enzymolysis of potato protein^18^ are among several known examples. Cebrián-García et al.^19^ studied the ultrasound-assisted esterification of valeric acid to alkyl valerates promoted by biosilicified lipases. Biosilicified enzymes had slightly improved specific activities and product yields for the ultrasound-assisted esterification compared to the free enzyme in ethanol.

Using acoustic waves on enzymes causes increase in the mass transfer rate of the reagents to the active site^20^. Acoustic cavitation influence protein conformation, unfolding the polypeptide chain and reveling the active site^21^. Consequently, ultrasound can assist the enzymes contact with substrates^16^. In the reaction mixture, the shear forces caused by mechanical effects of ultrasound intensify the mass transfer, leading to the decrease of diffusion barrier^22^. Several research studies prove that ultrasound affects differently distinct enzymes, each having a specific tolerance and sensitivity to ultrasound. In order to reach the maximum value of catalytic activity the optimum ultrasonic conditions must be determined, known being that high power ultrasound ends up in enzymes denaturation^23,24^. Often, a low intensity, short duration ultrasonic treatment is more likely to enhance enzyme activity, while prolonged exposure may lead to loss of stability and decrease in enzyme activity^20,25^.

In our previous paper^26^ the bio-catalyzed ultrasound assisted synthesis of isoamyl acetate was reported, and another feature of ultrasound, namely unlocking the Lipozyme 435 enzyme for a specific enzymatic esterification was described.

The main objective of this paper is to develop new data regarding the biocatalytic effect of Lipozyme 435 under ultrasound-assisted esterification of acetic acid with isoamyl alcohol. The novelty of this paper is the intensification of the enzymatic esterification process using a continuous loop reactor. The thermodynamics involved in the enzymatic esterification was also evaluated following the influence of process conditions on the reaction rate, monitoring some of the reaction’s parameters such as temperature and residence time. With these new data in hands, the best sonication conditions (ultrasonic power and duty cycle) and the reusability of enzymes, for this particular enzymatic process were established.

## Materials and methods

### Materials

Acetic acid (analytical grade, 99.99% purity) was purchased from Chemical Company CHIMOPAR SRL, Romania. Isoamyl alcohol (≥ 98%) was provided by Sigma-Aldrich. The commercial enzyme used as catalyst was Lipozyme 435 provided by curtesy of Novozyme, Denmark, as a *Candida antarctica* lipase immobilized on a macroporous anion exchange resin. The enzyme support, Duolite A 568 (Duolite International SA, Paris), is a porous granular, weak base anion exchange resin based on a cross-linked phenol–formaldehyde polycondensate with a hydrophilic structure and controlled pore size distribution. Acetone was purchased from Chemical Company Chimreactiv SRL, Romania.

### Experimental setup

The esterification reaction was carried out in a loop reactor^26,27^ described in our previous work. The advantage of using a loop reactor is to minimize cavitation activity and maximize mass transfer^26^. The esterification reactions carried out at 50 °C with various flow rates and an acid-to-alcohol molar ratio of 1:2. The total volume of esterification mixture was 5 mL and it was maintained constant for all reactions. Sonication was performed using a probe system (Vibracell VCX-750), with a power supply setting at various amplitude (20–30%). The samples were taken at certain reaction times and analyzed by gas chromatography. All the experiments were carried in triplicates to check the reproducibility of the results and certainly the obtained data were reproducible and the experimental errors were less than ± 3% of the reported average values of the results. Results from tables snd figures are expressed as mean ± standard deviation of the values obtained in triplicates.

### Gas chromatography analysis (GC)

Quantitative analysis of the esters was performed using an HP 6890 gas chromatograph equipped with flame ionization detector (FID). The column used was a HP-INNOWAX 19091N-133 cross-linked PEG with 30 m × 250 µm.The oven was set to heat the column from 50 to 250 °C under a heating gradient of 10 °C/min. Helium was the carrier gas (flow rate 1 mL/min) and *n*-butanol was used as an internal standard for the ester concentration. The samples were analyzed in duplicates.

### Kinetics of esterification process

The reaction between acetic acid and isoamyl alcohol is an equilibrium-limited reversible reaction:
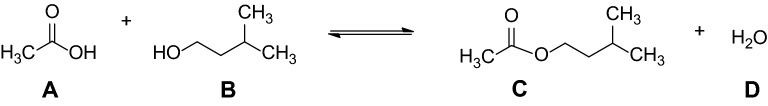


An isoamyl alcohol excess was used to drive the reaction toward the product’s side. The activation energy for the conventional reaction and the ultrasound assisted reaction were calculated. In order to assess the activation energy, the rate constant was determined from the general reaction rate equation:

1 $$-{r}_{A}= \frac{\partial {C}_{A}}{\partial t}=\frac{\partial {C}_{B}}{\partial t}=k{C}_{A}{C}_{B}{\gamma }_{A}{\gamma }_{B}$$

where: γ_A_ and γ_B_ are activity coefficients, C_A_ and C_B_ are concentrations of the reagents during the reaction and k is rate constant.

It is assumed that the concentration of reagents depends on the initial concentrations (C_A0_ and C_B0_) and each component fraction (X_A_ and X_B_) and the reaction rate can be rewritten as follow:

2 $$-{r}_{A}={C}_{A0} \frac{\partial {X}_{A}}{\partial t}=k{(C}_{A0}-{C}_{A0}{X}_{A}){(C}_{B0}-{{C}_{A0}{X}_{A}) {\gamma }_{A}\gamma }_{B}$$

The initial molar ratio is $M=\frac{{C}_{B0}}{{C}_{A0}}$. For an ideal system, γ_A_ and γ_B_ are considered constants, so the logarithm of Eq. (2) can be rearranged to yield the reaction rate constant in Eq. (3)^28^.

3 $$n\frac{1-{X}_{B}}{1-{X}_{A}}=ln\frac{M-{X}_{A}}{M(1-{X}_{A})}=ln\frac{{C}_{B}{C}_{A0}}{{C}_{B0}{C}_{A}}=ln\frac{{C}_{B}}{{MC}_{A}}=(M-1){C}_{A0}kt$$

Several experiments have been carried out to analyze the influence of temperature, for both conventional heating and ultrasound assisted reactions. The temperature dependency of esterification reaction was determined by calculating the activation energy (*E*_*a*_) at different temperature levels. For this, the *Arrhenius Law* was used:

4 $$k=A{e}^{\frac{-Ea}{RT}}$$

where: *E*_*a*_ is the activation energy, A is pre-exponential factor, R is the universal gas constant (8.314 J/mol K), and T is the absolute temperature.

The equilibrium concentration of the ester was determined considering a value of 4.9 for the equilibrium constant^29^. The equilibrium concentration of ester (C_eq_) for our reaction is 477 mg/g_mixture_, and the maximum ester concentration (c_max_) for isoamyl acetate is 551 mg/g_mixture_ (for a molar ratio alcohol:acid = 2:1).

### Enzyme reusability

Enzyme reuse requires a washing step before the next esterification reaction. For this, at the end of each enzymatic esterification, the immobilized lipase was washed with acetone for 10 min, at room temperature. After removing the solvent, the lipase was dried in an air current flow (previously passed through an active charcoal filter) at 50 °C for 15 min and reused in a new reaction.

### Determination of ultrasonic power by calorimetric method

Several methods are available to estimate the amount of ultrasound power that has entered a sonochemical reaction^30,31^. Calorimetry is the most used method, and it involves measuring the initial rise of temperature produced when a system is irradiated by acoustic power. This assumes that almost all mechanical energy produces heat and thus the output power can be obtained by calorimetry. The temperature rise of a fixed amount of reaction mixture, in an isolated glass reactor and in the coupling fluid, at a given time, was measured. Using this information, the energy dissipated in the liquid, inside the reactor, and in the coupling fluid, was calculated (Table 1) using Eqs. (5) and (6).

**Table 1:** Ultrasonic power absorbed in the reactor and in the coupling fluid and ultrasonic power density.

Ultrasonic amplitude, %	Ultrasonic power, W		Ultrasonic power density, W/mL	
	In reactor	In coupling fluid	In reactor	In coupling fluid
20	0.032 ± 0.002	12 ± 1.0	0.053 ± 0.0003	0.103 ± 0.008
25	0.044 ± 0.004	17 ± 1.8	0.073 ± 0.0006	0.144 ± 0.015
30	0.068 ± 0.0045	27 ± 1.6	0.114 ± 0.00075	0.227 ± 0.013

5 $$Q=m \times {c}_{p} \times \Delta T$$

6 $$P=\frac{Q}{t}$$

where Q is the quantity of heat (joule), ΔT is the temperature variation (°C), m is the mass of fluid (kg) and c_p_ (kg/°C ) is the specific heat, P is the ultrasonic power (W) and t (seconds) is the time in which the temperature rise was measured.

It is observed that as the amplitude of the applied ultrasound increases, the difference between the power absorbed in the reactor and the power absorbed in the coupling fluid is increasing.

## Results and discussion

### The influence of ultrasonic power

**Figure 1:**
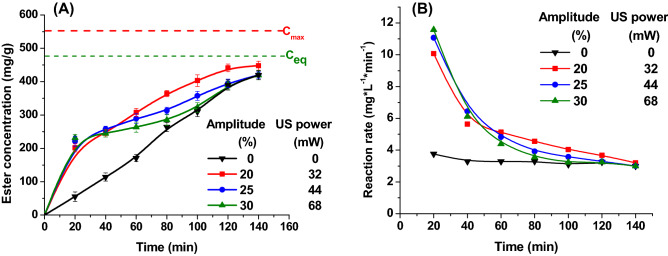
The influence of ultrasound amplitude on the ester concentration (**A**) and on the reaction rate (**B**) (reaction conditions: temperature 50 °C, flow rate 0.16 mL/min).

To study the effect of the ultrasound power, experiments were performed by varying the amplitude from 20 to 35% to find the best power needed in order to achieve an efficient cavitation generation to perform esterification without damaging the enzyme. With the increase of amplitude, the energy input in the reaction system increases, too, leading to a higher number of cavitation bubbles and therefore to more chances to damage the enzyme. The amplitudes over 40% physically damage the enzymes. The amplitude together with the duty cycle and sonication time are parameters showing how the ultrasonic power could influences the enzyme and enzymatic activity. Ultrasounds can increase enzymatic catalytic rate by two phenomena, either by increasing mass transfer or by directly influencing the enzyme^32^. Both effects were followed in the experimental program. At the beginning, the power of the continuously applied ultrasound was changed by rising the amplitude from 20 to 30%. As shown in Fig. 1, ultrasound causes a significant increase in the reaction rate in the first 20 min, but later it is found that at high powers (44–68 mW) an inhibition of enzyme activity occurs and consequently the reaction rates decrease. Thus, the 20% amplitude was considered optimal and it was used for the following studies.

### The influence of duty cycle

**Figure 2:**
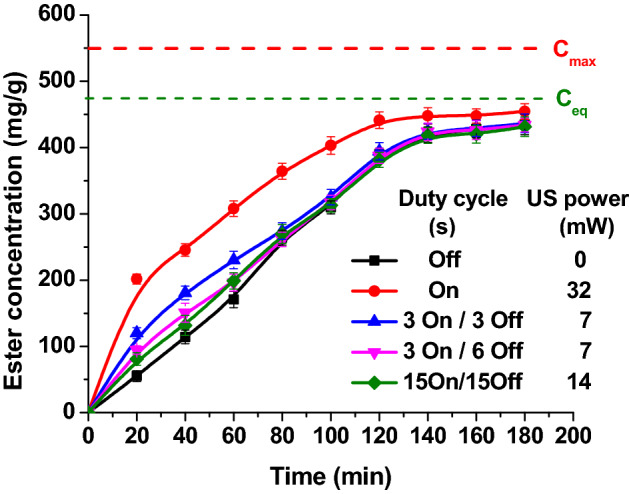
The influence of the sonication power on the ester concentration (reaction conditions: temperature 50 °C, flow rate 0.16 mL/min, ultrasonication time 3 h, ultrasound amplitude 20%).

**Figure 3:**
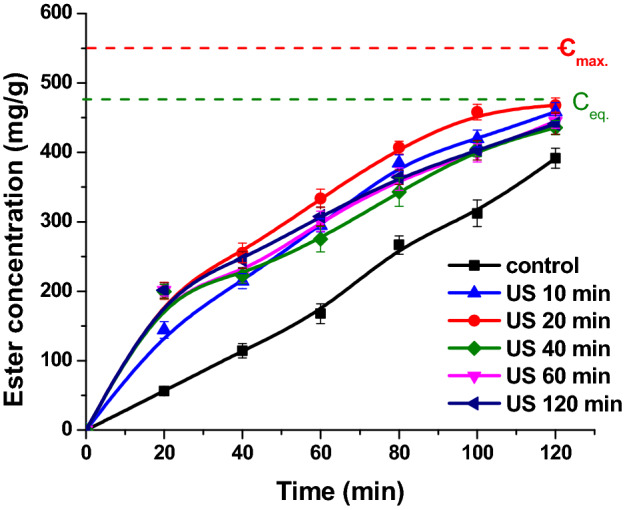
The influence of the sonication time on the ester concentration (reaction conditions: temperature 50 °C, acid to alcohol molar ratio 1:2, flow rate 0.16 mL/min, continuous ultrasonication, power 20 W, amplitude 20%).

The power is a very important parameter for the ultrasonic assisted reactions. For classical esterification reactions, higher ultrasound power gives higher conversions^33^. In case of enzyme catalyzed reaction, ultrasounds may lead to improvement in enzyme activity^34^. High ultrasonic powers may deactivate, or even denature the enzyme^35^. A series of experiments were performed to identify the optimum required ultrasound power for enzyme activation. In order to modify the ultrasonic power, instead of continuously sonication, the acoustic duty cycle was used and the results are shown in Figs. 2 and 3. However, the use of the duty cycle did not lead to better results than the continuous use of ultrasound with an amplitude of 20% regardless of the flow rate used.

These experimental data showed that the use of pulse ultrasound mode (3 s on/3 s off) gives almost similar results as for continuous sonication using less grid power (half), as seen in Table 2. Even if the ester concentration is higher for continuous sonication, the difference is low, only 4.4%, this could be considered an advantage of pulse sonication.

**Table 2:** Different powers applied to the system.

Amplitude, %	Grid power, W	Power absorbed in the reactor (determined by calorimetry), W	Power read from Vibracell, W
off	22	0	0
20	60	0.032	20
25	73	0.044	30
30	86	0.068	43
20% (3 s on /3 s off)	30	0.016	10

### The influence of the sonication time

In literature, it is reported that long-time exposure to ultrasound at low temperature leads to the enzyme inactivation by splitting of low molecular weight polypeptides or individual amino acids and the oxidative mechanisms^36^. Ultrasonic shear stresses generated by acoustic cavitation can degrade polymers of high molecular mass and promotes enzyme denaturation^37^. Wang et al.^38^, studied the effects of low intensity ultrasound on cellulase pretreatment, noticing that the highest activity of immobilized cellulase was reached when the sample was treated with ultrasounds, 24 kHz and 60 W US power, for 10 min. Using scanning electron microscopy (SEM) they proved that the ultrasonic treatment lead to an increase in the surface area of the immobilized cellulase. When the enzyme was treated with ultrasounds for a short period of time activity of the enzyme was increased by 24.67% over the control.

To establish optimal sonication time for Lipozyme 435 experiments at different continuous ultrasound exposures: 10, 20, 40 and 60 min were performed, as shown in Fig. 3. It can be noticed that the best results were obtained when ultrasounds were applied for 20 min, the ester concentration increased with 27.4%. The possible reason for this could be that applying ultrasound for 20 min provides enough energy for the activation of enzyme, and better exposure of enzymatic active sites, without denaturing Lipozyme 435 enzyme. 10 min ultrasonic treatment is not enough for the enzyme activation, and 40 and 60 min are too much, the ultrasound starting to affect physically Lipozyme 435 enzyme.

### Activation energy measurements

**Figure 4:**
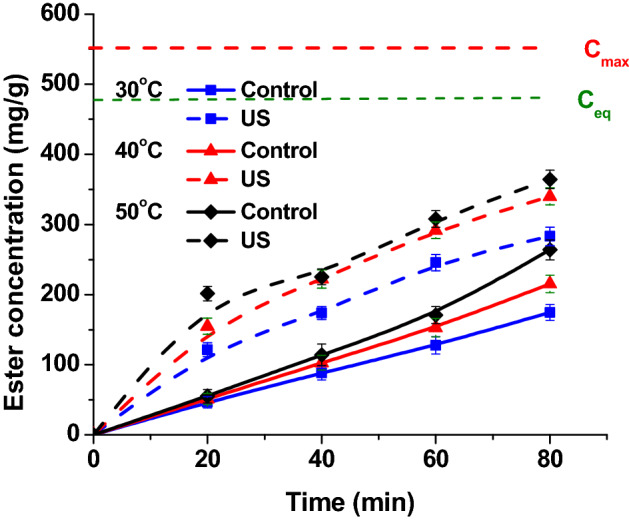
The influence of temperature on the ester concentration for control and ultrasound assisted esterification (reaction conditions: flow rate 0.16 mL/min, continuous ultrasonication, power 20 W, amplitude 20%).

To determine the apparent activation energy for conventional and for ultrasonically assisted process, a series of enzymatic esterification reactions has been performed at different reaction temperatures: 30, 40 and 50 °C. As shown in Fig. 4, it is obvious that ester concentration increases with increasing temperature. The effect of ultrasound is obvious, ester concentrations are significantly higher in the case of ultrasound-assisted reactions.

**Figure 5:**
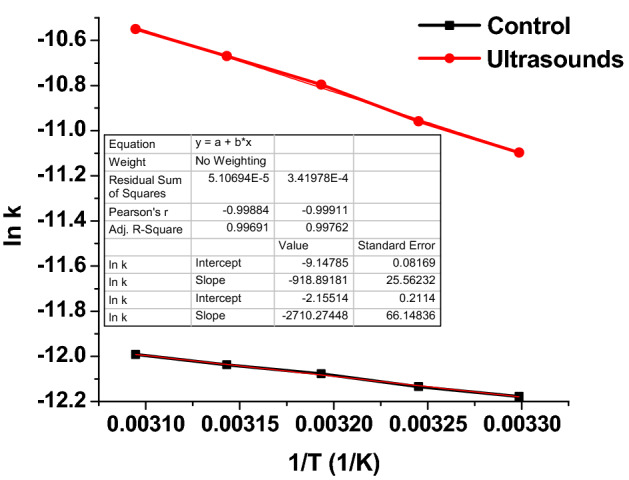
Arrhenius plot of effective reaction rates for control and ultrasound assisted esterification (reaction conditions: temperature 50 °C, flow rate 0.16 mL/min, continuous ultrasonication, power 20 W, amplitude 20%).

The activation energy for each esterification reaction can be determined by plotting the logarithm from rate constant versus the reciprocal of absolute temperature as shown in Fig. 5. The calculated activation energies for both reactions are presented in Table 3.

**Table 3:** The activation energy for control and ultrasound assisted enzymatic esterification calculated using Arrhenius diagram.

Reaction conditions	Slope, K	E_a_, J/mol
Control	− 918.892	7640.1
Ultrasound	− 2710.27	22,534.6

The apparent activation energy (E_a_) for the synthesis of isoamyl acetate in the presence of ion-exchange resins determined in literature was in the range 42–45 kJ/mol^39,40^. Enzymes are biocatalysts that lower the activation energy (E_a_) that is required to convert the substrates to products^41^. However, immobilizing the enzyme, diffusion may become rate limiting and the reaction may switch from kinetic to diffusion controlled resulting in a change in the measured apparent E_a_.

From the analysis of the data presented in Table 3 it can be noticed that the apparent activation energies in the absence of ultrasound is very low, which means that the process takes place in diffusion mode. When ultrasound is applied, the apparent activation energy increases greatly, indicating the shift to the kinetic regime. This value is consistent with those determined for esterification reactions in the presence of enzymes immobilized in organogel agar. The diffusion study has revealed that the structure of these organogels does not provide a barrier for the substrates to enter the catalyst^41^.

### The influence of flow rate

**Figure 6:**
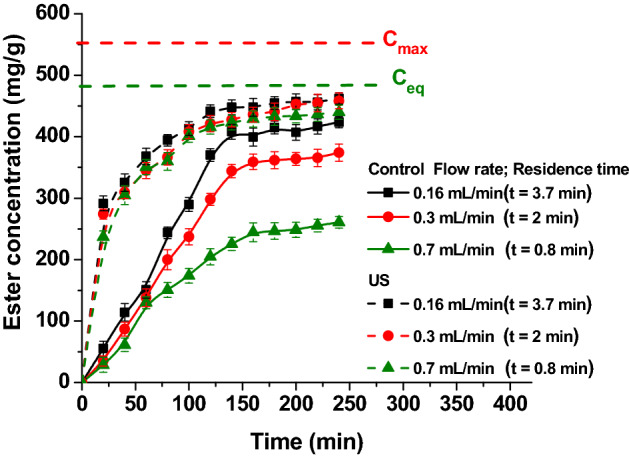
The influence of flow rate on ester concentration, by conventional and ultrasound assisted processes (reaction conditions: temperature 50 °C, continuous ultrasonication, power 20 W, amplitude 20%).

The flow rate is important for the process of continuous esterification reactions. Lower values of residence time determine higher productivity. For this study three different reactants flow rates: 0.16, 0.3 and 0.7 mL/min where used. The results are presented in Fig. 6.

For conventionally carried out reactions the flow rate is very important. Lower flow rates (0.16 mL/min) assure longer contact time (3.7 min) between the reactants involved and the enzyme, resulting in a more efficient conversion.

Ultrasound assisted reactions lead to a higher isoamyl acetate concentration, for all three flow rates. The most important effect of ultrasound is observed when the flow rate is lower, in these conditions the combined effect of longer residence time and that of ultrasound determines the decrease of the diffusion resistance even more and consequently the increase of the conversion. But, the difference between the ester concentrations obtained by ultrasound assisted esterification at all flow rates is not higher. This behavior can be explained by mass transfer considerations. At higher flow rates, the mass transfer residences in the liquid phase is reduced by the turbulences generated by the bubbles inside the reactor. At higher flow rates, the regents mixing is more vigorous and intense, resulting in a more efficient conversion.

### The enzyme reusability

One of the most important advantages when using immobilized enzymes is the prospect of their reusability, a crucial economic and environmental aspect for employing enzymes in industrial applications. The reusability and recovery of an enzyme is of high priority in order to decide if the bioprocess is viable or not. In order to evaluate the potential of immobilized lipase for industrial enzymatic esterification application, the stability of Lipozyme 435 was deduced by measuring the concentration of isoamyl acetate from successive esterification reaction under optimal conditions. The immobilized enzyme used, Lipozyme 435, after the enzyme was further washed with acetone and dried in a warm air flow and reused. Washing with acetone is a crucial step to remove accumulation of product and acid content from the environment of the immobilized biocatalyst. The residue of acid and isoamyl ester may block active sites of lipase and hinder the next successive reaction cycles yielding lower ester concentrations^42^.

**Figure 7:**
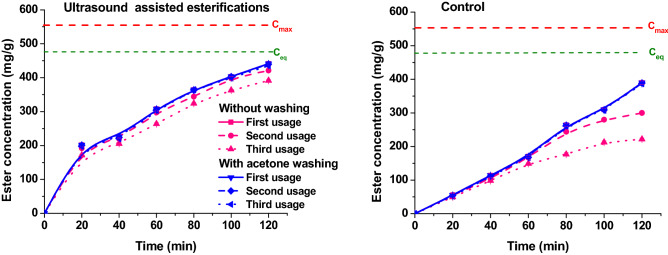
The influence of washing with acetone on the reusability of immobilized Lipozyme 435 for repeated cycles. by conventional process (reaction conditions: temperature 50 °C) and ultrasound assisted process (continuous ultrasonication, power 20 W, amplitude 20%).

The use of Lipozyme 435 enzyme in three consecutive reactions, without acetone washing after each reaction results in an isoamyl concentration decrease. This decrease in the enzyme activity was noticed in both reactions with conventional heating and ultrasound assisted reactions, as presented in Fig. 7. The possible reason for this could be the blockage of Lipozyme 435 active sites due to the retention of some reactants or/and product (especially acetic acid) or the breakdown of Lipozyme 435 beads during the reaction, as a consequence of mechanical effects of ultrasound^43^.

The isoamyl acetate concentration in conventional heating reaction with acetone washing, after three consecutive uses, lowers with 1%, comparative with the reactions without acetone washing, were the isoamyl ester concentration drops with 43.33%. For the ultrasound assisted reaction, the ester concentration drop without washing was of 11.33%. Washing with acetone after each reaction, the ester concentration drop after three consecutive reactions was only 1.36%.

Thus, these results clearly show significant improvement in Lipozyme 435 reusability and regeneration when using the acetone washing method.

## Conclusion

The esterification process of acetic acid with isoamyl alcohol catalyzed by the commercial enzyme Lipozyme 435 was carried out. The best sonication conditions to improve Lipozyme 435 activity, stability and reusability were determined. The results indicate that the maximum ester concentration was obtained for ultrasound-assisted process at temperature 50 °C, flow rate 0.16 mL/min. The best ultrasonication conditions are: continuously applied sonication for a short time (20 min), ultrasound power 32 mW, and amplitude 20%. The results show a favorable perspective to improve the esterification efficiency and reduce the reaction time, using ultrasounds for short time periods. The calculated activation energy for the synthesis of isoamyl acetate is 7640.1 J/mol for conventional esterification and 22,534.6 J/mol for ultrasound-assisted reaction. In this enzymatic esterification, the enzyme can be reused several times after regeneration by washing with acetone, making the process cost effective. The results achieved in this research can be used for scaling up the process at higher level.
